# Targeting a novel cancer-driving protein (LAPTM4B-35) by a small molecule (ETS) to inhibit cancer growth and metastasis

**DOI:** 10.18632/oncotarget.11325

**Published:** 2016-08-17

**Authors:** Maojin Li, Rouli Zhou, Yi Shan, Li Li, Lin Wang, Gang Liu

**Affiliations:** ^1^ Department of Cell Biology, School of Basic Medical Sciences, Peking University, Haidian District, Beijing 100191, China; ^2^ School of Pharmaceutical Sciences, Tsinghua University, Beijing 100084, P.R. China; ^3^ Department of Synthetic Medicinal Chemistry, Institute of Materia Medica, Chinese Academy of Medical Sciences and Peking Union Medical College, Beijing 100050, China

**Keywords:** LAPTM4B-35, ethylglyoxal bisthiosemicarbazon (ETS), hepatocellular-carcinoma, cancer targeted therapy, apoptosis

## Abstract

Our previous studies demonstrated that LAPTM4B-35 is overexpressed in a variety of solid cancers including hepatocellular carcinoma (HCC), and is an independent factor for prognosis. LAPTM4B-35 overexpression causes carcinogenesis and enhances cancer growth, metastasis and multidrug resistance, and thus may be a candidate for therapeutic targeting. The present study shows ethylglyoxal bisthiosemicarbazon (ETS) has effective anticancer activity through LAPTM4B-35 targeting. Bel-7402 and HepG2 cell lines from human HCC were used as cell models in which LAPTM4B-35 is highly expressed, and a human fetal liver cell line was used as a control. The results showed ETS has a specific and pronounced lethal effect on HCC cells, but not on fetal liver cells in culture. ETS also attenuated growth and metastasis of human HCC xenograft in nude mice, and extended the life span of mice with HCC. ETS induced HCC cell apoptosis, and upregulated a large number of proapoptotic genes and downregulated antiapoptotic genes. When endogenous overexpression of LAPTM4B-35 was knocked down with RNAi, the killing effect of ETS on HepG2 cells was significantly attenuated. ETS also inhibited phosphorylation of LAPTM4B-35 Tyr_285_, which involves in activation of the PI3K/Akt signaling pathway induced by LAPTM4B-35 overexpression. In addition, the induction of alterations in quantity of c-Myc, Bcl-2, Bax, cyclinD1 and Akt-p molecules in HepG2 cells by LAPTM4B-35 overexpression could be reversed by ETS. Conclusion: ETS is a promising candidate for treatment of HCC through LAPTM4B-35 protein targeting.

## INTRODUCTION

LAPTM4B-35 (NP_060877) is encoded by *Lysosomal protein transmembrane 4 beta* (*LAPTM4B*, NM_018407), which was first cloned and found to be a driver gene of hepatocellular carcinoma (HCC) in our laboratory [1, 2]. LAPTM4B-35 is overexpressed in a wide variety of solid cancers, including HCC [2–4], lung cancer [5, 6], etc., as well as a variety of cancer cell lines [7]. The elevation of LAPTM4B-35 was associated with pathological grade, high tumor recurrence, metastasis and poor postoperative survival, suggesting that LAPTM4B-35 is an independent prognostic factor [2–6, 8, 9]. Overexpression of LAPTM4B-35 promotes antiapoptosis, proliferation, migration, invasion [10, 11] and multidrug resistance of cancer cells [12, 13], and it also elicits oncogenesis of NIH 3T3 cells from mouse fibroblast cell line [14] and L02 cells from normal human liver cell line [15]. Overexpression of LAPTM4B-35 upregulates expression of a number of oncogenes, and downregulates expression of some tumor suppressing genes [4, 10, 11]. In addition, the carcinogenesis-promoting PI3K/Akt signaling pathway is over-activated by LAPTM4B-35 overexpression [12, 15]. Conversely, knockdown of endogenous LAPTM4B-35 expression with RNAi reverses all of these malignant cellular and molecular phenotypes *in vitro*, and inhibits tumor growth and metastasis of human HCC xenograft in nude mice [11]. It thus has been proposed that *LAPTM4B* is a cancer driver gene, and LAPTM4B-35 is an oncoprotein. Therefore, LAPTM4B-35 may be a novel molecular target for treatment of HCC and other solid cancers.

Given that overexpression and/or hyper-activation of EGFR are associated with oncogenesis and poor prognosis in many cancers. Recently, it was found that LAPTM4B can bind to EGFR, and subsequently enhance and prolong the EGFR signaling [16] and initiate autophagy through a non-canonical EGFR signaling pathway and trafficking [17], which both facilitate the functions of EGFR on promoting cancer cell survival and proliferation. Therefore, LAPTM4B is associated with EGFR in oncogenesis and progression. It is well known that EGFR is a rational target for cancer therapy. However, inhibitors that target canonical, ligand-stimulated EGFR signaling have proven to be largely ineffective in treating many EGFR-dependent cancers with the exception of non-small cell lung cancers (NSCLC) carrying activating mutations in EGFR. If targeting EGFR in combination with LAPTM4B in carcinoma targeted therapy, an improved outcome would be expected.

In this study small synthetic chemical compounds with anticancer activity were screened for targeting LAPTM4B-35. A total of 1697 compounds were tested for killing effect on HCC cells. We found that ethylglyoxal bisthiosemicarbazon (ETS) has significant antitumor activity *in vitro* and *in vivo* by LAPTM4B-35 protein targeting.

ETS was first synthesized in the 1950's for therapeutic use against helminth or parasites [18] and was subsequently found to have anti-sarcoma (S-180) activity in mice [19]. However, the specific mechanism of ETS has not been fully determined. We have confirmed that ETS has lethal activity against a wide range of human cancer cell lines, such as HepG2, Bel-7402, HLE and HeLa. We also found that ETS inhibits the PI3K/AKT signaling pathway by suppressing phosphorylation of Tyr_285_ in the C-terminus of LAPTM4B-35 protein, which reduces interaction of LAPTM4B-35 and PI3K p85α, and thus inhibits phosphorylation/activation of Akt. As a result, the molecular and cellular malignant phenotypes, which are enhanced by overexpression of LAPTM4B-35, are inhibited by ETS. ETS is therefore a candidate for treatment of HCC and some other cancers in which LAPTM4B-35 overexpresses.

## RESULTS AND DISCUSSION

### Screen of cancer-inhibiting small molecules

1697 synthetic small molecules in stock in our library were screened. Among them 12 hits were shown to have an effective inhibitory effect on Bel-7402 and HepG2 HCC cell lines in a dose- and time-dependent manner. Out of the 12 hits, IMMLG-597 and the three derivatives (WL-07-5, WL-07-19 and WL-07-21), which have similar structures and are categorized as bisthiosemicarbazons, showed the strongest inhibitory effect on cancer cells and transformed cells. Among them IMMLG-597 showed relatively stronger activity (lower IC_50_) than the other three derivatives (data not shown). Therefore, it was chosen to study the anticancer effects and the mechanisms. The chemical structure of IMMLG-597 is ethylglyoxal bis-thiosemicarbazone and abbreviated as ETS. Its structure is as follows:


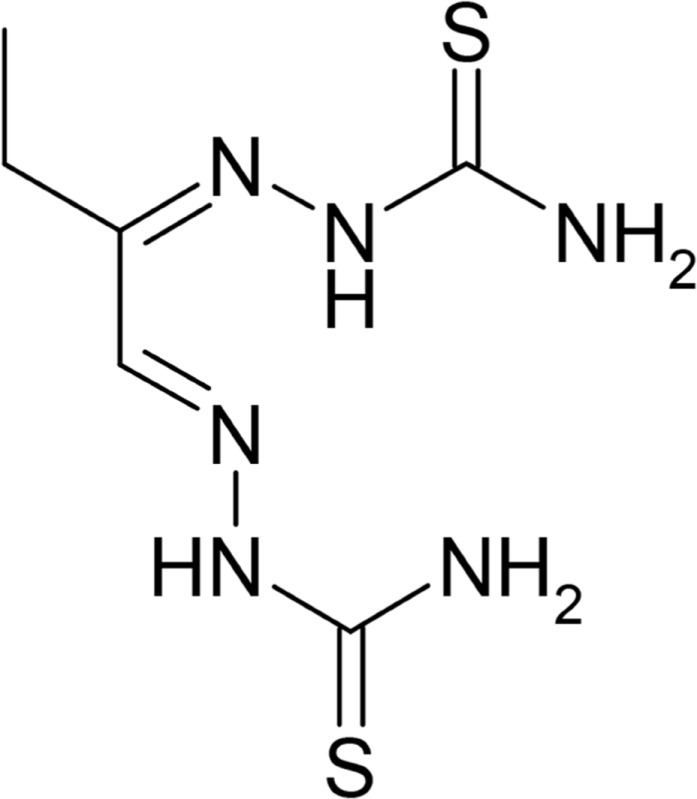


### ETS (IMMLG-597) inhibits/kills cancer cells

**Figure 1:**
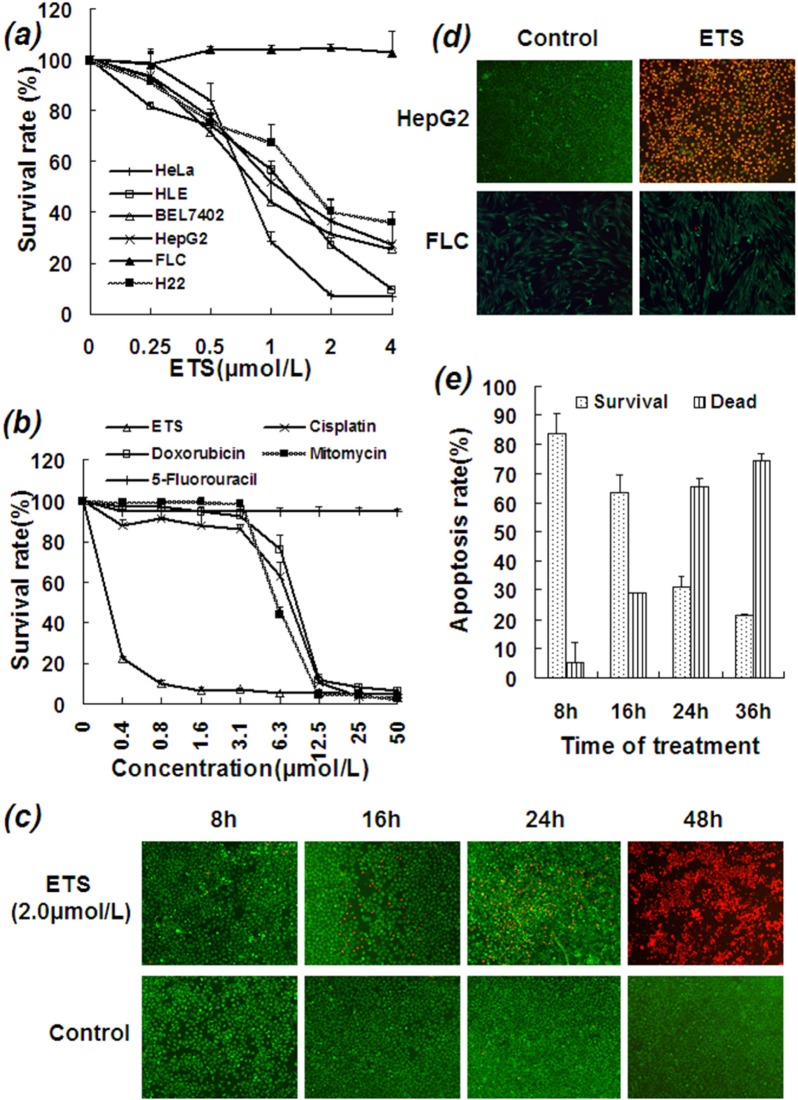
Inhibitory and killing effects of ETS on HCC cells **a.** Various cancer cell lines were cultured in the absence or presence of ETS at indicated concentrations for 48 h. **b.** HepG2 cells were cultured in the absence or presence of various drugs at indicated concentrations for 48 h. **c.** HepG2 cells were cultured in the presence and absence of ETS for indicated hours and then treated with LIVE/DEAD* Viability/Cytotoxicity Kit for detection of viable and dead cells. The killing effect of ETS is time-dependent with the majority of HepG2 cells were killed by ETS at 48 hours. **d.** The cells were fluorescently double-stained with Calcein-AM (1 μmol/L) and EthD-1 (2 μmol/L) at 37°C for 30 min and then surveyed under fluorescence microscope. Cells emitting green fluorescence were viable cells which were merely stained by Calcein-AM. Cells emitting red fluorescence were dead cells or apoptotic cells which merely stained by EthD-1. Upper panel: HepG2 HCC cells were treated with ETS at a concentration of 2 μmol/L for 48 h. The vast majority of HepG2 cells were killed by ETS. Lower panel: human fetal liver cells were treated with ETS at a concentration of 25 μmol/L for 48 h. None of fetal liver cells were killed by ETS. **e.** HepG2 cells were treated with ETS at a concentration of 2 μmol/L for 8, 16, 24 or 36 hours. The apoptotic cells were measured by Flow-cytometry. The dying cells including at early and late apoptotic phases were calculated together. The promoting effect of ETS on apoptosis of HepG2 cells was time-dependent. The experiment was repeated several times.

Cancer cell growth curves showed that ETS significantly reduced survival rates in a number of cancer cell lines, including HepG2 (IC_50_: 0.9 μmol/L), Bel-7402 (IC_50_: 0.7 μmol/L), HeLa (IC_50_: 0.6 μmol/L), HLE (IC_50_: 1.1 μmol/L), and H22 (IC_50_: 1.6 μmol/L) (Figure 1a). In the series of hepatocellular carcinoma cell lines, Bel-7402 and HepG2 cells both express LAPTM4B-35 at very high levels and were very sensitive to ETS; HLE cells express LAPTM4B-35 at a relatively low level [11] and were less sensitive to ETS. Importantly, Figure 1a also showed that, ETS had little inhibitory effect on normal fetal liver cells (FLC) which express LAPTM4B-35 at a low level. This was also the case even at a concentration of ETS that was 200 times greater than those which had an inhibitory effect on HCC cells (data not shown). At the same time, the inhibitory effect of ETS at lower concentrations (less than 6.3 μmol/L) on HepG2 cells was much stronger than that of Cisplatin, Doxorubicin, Mitomycin or 5-Fluorouracil *in vitro* (Figure 1b). Additionally, Calcein-AM/EthD-1 fluorescence double staining assays showed a time-dependent lethal effect of ETS on HepG2 cells (Figure 1c). In this assay HepG2 cells were fluorescently double-stained with Calcein-AM (1 μmol/L) and EthD-1 (2 μmol/L) at 37°C for indicated times in the presence and absence of ETS (2 μmol/L). Cells showing green fluorescence were viable cells stained by Calcein-AM, and cells showing red fluorescence were dead or apoptotic cells stained by EthD-1. Treatment of ETS at a concentration of 2 μmol/L for 48 hours resulted in death of almost all HepG2 cells, whereas human fetal liver cells survive even after treatment at a concentration up to 25 μmol/L for 48 hours (Figure 1d). These results demonstrate the selectivity of this lethal effect on cancer cells of ETS.

To evaluate the cellular mechanism by which ETS kills HCC cells, apoptosis was detected at the cellular, molecular, and gene levels. Flow cytometry analysis showed that ETS (2 μmol/L) induces apoptosis in HepG2 cells in a time-dependent manner (Figure 1e). For example, 10.1% of cells were dead after 8 h, 15.8% after 16 h, 29.1% after 24 h, 63.0% after 36 h, and ~100% at 48 h. The apoptotic rate counts included all cells in early and late apoptotic phases.

### ETS significantly inhibits growth and metastasis of human HCC xenograft *in nude mice*, and extends the life span of mice with HCC ascites

**Figure 2:**
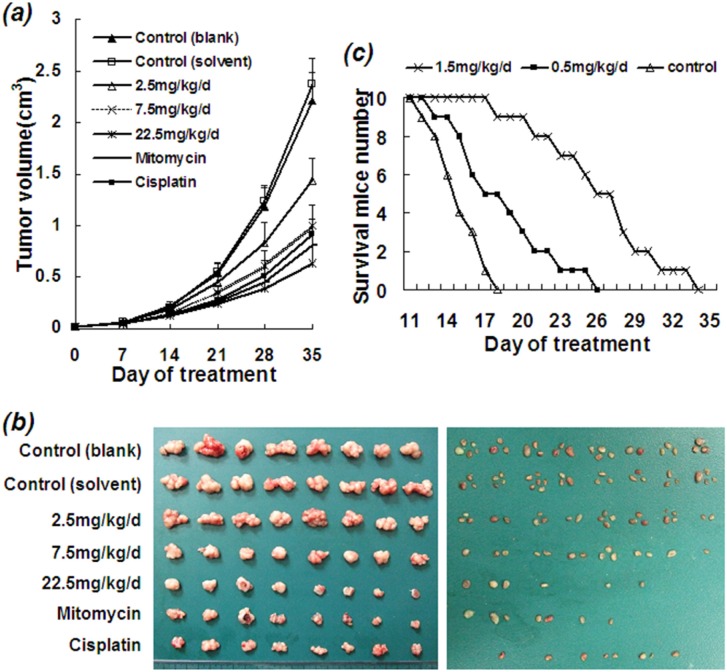
Inhibitory effect of ETS on growth and metastasis of human HCC xenograft in nude mice Human HCC Bel-7402 cells (1 × 10^6^) were subcutaneously inoculated into each nude mouse. ETS (2.5, 7.5, 22.5 mg/kg/d), cisplatin (1.0 mg/kg/d), mitomycin (1.0 mg/kg/d), solvent and PBS (controls) were administered by intraperitoneal injection from day 9 when the xenograft grew out. Mitomycin and cisplatin were used as the positive controls, solvent and PBS were used as negative controls. The inhibitory effect of ETS on xenograft growth was observed to be dose-dependent as compared with the control groups of solvent and PBS. **a.** Tumor growth curves of human HCC xenograft in nude mice with variant treatments. **b.** Tumor photograph of human HCC xenograft grown in nude mice with variant treatments for 6 weeks. Left panel: Size of human HCC xenograft in variant groups. Right panel: Number of lymph nodes metastases in variant groups. **c.** The survival curves of mice with ascites HCC in variant groups. Mouse hepatocellular carcinoma H22 cells (1 X10^6^) were inoculated into peritoneal of each ICR mouse. ETS (0.5, 1.5 mg/kg/d) and solvent were intraperitoneally administered to each ICR mouse in various group (*n* = 10). The life span showed a significant prolongation with a dose-dependent manner in the ETS groups.

ETS showed a significant inhibitory effect on HCC growth and metastasis *in vivo*. Human HCC BEL-7402 cells were subcutaneously inoculated on the upper back, and ETS was then administered either by intra-tumor or intraperitoneal injection. Either way of ETS injection inhibited HCC xenograft growth. The effect of ETS on growth and metastasis of human HCC xenograft in nude mice is shown in Table 1 and Figure 2.

**Table 1:** The inhibitory efficacy of ETS administered by intraperitoneal injection on the xenograft of human HCC in nude mice

Group	Mice (No.)	Tumor weight (g,$\bar{x}\pm s$)	Inhibitory rate (%)	Metastatic lymph nodes (No., $\bar{x}\pm s$)
Control (PBS)	8	1.960 ± 0.133	0	3.3 ± 0.89
Control (solvent)	8	2.073 ± 0.118	0	3.5 ± 1.07
ETS (2.5 mg/kg/d)	8	1.276 ± 0.104^*^	38.4%	2.8 ± 0.71
ETS (7.5 mg/kg/d)	8	0.794 ± 0.090^**^	61.7%	1.8 ± 0.71
ETS (22.5 mg/kg/d)	8	0.485 ± 0.123^**^^^^^	76.6%	0.8 ± 0.71^*^
Mitomycin (1.0 mg/kg/d)	8	0.673 ± 0.119^**^	67.5%	0.9 ± 0.83^*^
Cisplatin (1.0 mg/kg/d)	8	0.734 ± 0.098^**^	64.6%	1.0 ± 0.76^*^

* *P < 0.05*,

** *P< 0.01* vs. controls of PBS and solvent

^^ *P< 0.01* vs. group of mitomycin and cisplatin The nude mice were subcutaneously inoculated with human HCC Bel-7402 cells (1×10^6^) into the upper back, then stochasticly divided into 7 groups: ETS (2.5, 7.5, 22.5 mg/kg/d), mitomycin (1.0 mg/kg/d), cisplatin (1.0 mg/kg/d), solvent control and blank control (PBS), repectively. ETS, mitomycin and cisplatin were intraperitoneally administered by injection from the 9th day of inoculation when the tumor was palpable. The tumor weight and number of lymph node of metastases were measured at the experimental endpoint (6 weeks). The **i**nhibitory rate of tumor = (mean tumor weight of control - mean tumor weight of treated mice) / mean tumor weight of control × 100%. The results showed that the mean tumor weight of group of ETS (2.5, 7.5, 22.5 mg/kg/d) were much less than that of both solvent and PBS control groups (*P<0.05* for ETS (2.5 mg/kg/d) group, *P<0.01* for ETS (7.5 mg/kg/d) and ETS (22.5 mg/kg/d) group). Especially, the mean tumor weight of ETS (22.5 mg/kg/d) group was also less than that of mitomycin and cisplatin group (*P<0.01*). The number of metastatic lymph nodes in group of ETS (22.5 mg/kg/d) were much less than that of both solvent and PBS controls.

Growth and metastasis of xenograft from the human HCC cell line Bel-7402 in BALB\c-nude mice treated with ETS was significantly decreased, and the volume of tumor was notably reduced as compared with the solvent control and blank control (Figure 2a and 2b left panel). The number of metastatic axillary lymph nodes in mice treated with ETS was also obviously decreased as compared with the solvent and blank controls (Figure 2b right panel) (Table 1, *P<0.05*). The rate of inhibition of tumor in mice treated with ETS was 76.6% (at 22.5 mg/kg/d) (*P<0.01*), 61.7% (at 7.5 mg/kg/d) (*P<0.01*) and 38.4% (at 2.5 mg/kg/d) (*P<0.05*). The final mean tumor weight (g, $\bar{x}\pm s$) at week 6 after inoculation of HCC cells were 1.96±0.133 (blank control), 2.07±0.118 (solvent control), 1.28±0.104 (at 2.5 mg/kg/d), 0.79±0.090 (at 7.5 mg/kg/d), 0.49±0.123 (at 22.5 mg/kg/d), 0.67±0.119 (mitomycin), 0.73±0.098 (cisplatin) (Table 1), respectively; also indicating the inhibitory effect of ETS on tumor growth.

Particularly, the mean body weight of mice in group of ETS (7.5 mg/kg/d), ETS (2.5 mg/kg/d) and both controls including PBS and solvent at the end of test (6th week) increased, but decreased in group of ETS (22.5 mg/kg/d) and both mitomycin and cisplatin compared with that at the beginning of test (0 week) (Table 2). The mean body weight of mice in group of ETS (22.5 mg/kg/d), ETS (7.5 mg/kg/d) and ETS (2.5 mg/kg/d) at the end of test (6th week) were much more than that of mice in group of positive controls including mitomycin and cisplatin (*P<0.01*), which is to say, the body weight loss of mice treated with ETS is less than that of mice treated with mitomycin and cisplatin. Moreover, there were 2 (ETS (22.5 mg/kg/d)), 4 (mitomycin) and 3 (cisplatin) mice had died at the end of test, but had no died in group of ETS (7.5 mg/kg/d), ETS (2.5 mg/kg/d) and the negative controls including PBS and solvent, indicating ETS conferred less toxicity than mitomycin and cisplatin.

**Table 2:** The weight loss of mice treated with ETS was less than that of mice treated with cisplatin and mitomycin

Group	Mice (No.)	Weight of mice at the beginning of test (0 week, g)	Weight of mice at the end of test (6th week, g)	Weight change (g)
Control (PBS)	8	18.5±0.80	22.0±0.94	^+^ 3.5
Control (solvent)	8	18.5±0.88	21.0±1.02	+ 2.5
ETS (2.5 mg/kg/d)	8	19.2±0.86	20.3±0.98^^^^	+ 1.1
ETS (7.5 mg/kg/d)	8	18.9±0.80	19.1±1.01^^^^	+ 0.2
ETS (22.5 mg/kg/d)	8	19.0±0.86	17.8±0.92^^^^	^−^ 1.2
Mitomycin (1.0 mg/kg/d)	8	19.1±0.89	14.4±0.83	^−^ 4.7
Cisplatin (1.0 mg/kg/d)	8	19.1±0.83	15.5±0.87	^−^ 3.6

^^ *P< 0.01* vs. group of mitomycin and cisplatin

+ showed the mean body weight of mice increasing.

- showed the mean body weight of mice decreasing. The nude mice were subcutaneously inoculated with human HCC Bel-7402 cells (1×10^6^) into the upper back, then stochasticly divided into 7 groups: ETS (2.5, 7.5, 22.5 mg/kg/d), mitomycin (1.0 mg/kg/d), cisplatin (1.0 mg/kg/d), solvent control and blank control (PBS), repectively. ETS, mitomycin and cisplatin were intraperitoneally administered by injection from the 9th day of inoculation when the tumor was palpable. The mean body weights of mice in group of ETS (7.5 mg/kg/d), ETS (2.5 mg/kg/d) and both controls including PBS and solvent at the end of test (6th week) increased, but decreased in group of ETS (22.5 mg/kg/d) and both mitomycin and cisplatin compared with that at the beginning of test (0 week). The mean body weight of mice in group of ETS (22.5 mg/kg/d), ETS (7.5 mg/kg/d) and ETS (2.5 mg/kg/d) at the end of test (6 weeks) were much more than that of mice in group of positive controls including mitomycin and cisplatin (*P<0.01*), which is to say, the weight loss of mice treated with ETS is less than that of mice treated with mitomycin and cisplatin. Moreover, there were 2 (ETS (22.5 mg/kg/d)), 4 (mitomycin) and 3 (cisplatin) mice had died at the end of test, but had no died in group of ETS (7.5 mg/kg/d), ETS (2.5 mg/kg/d) and the negative controls including PBS and solvent.

In addition, the murine HCC H22 cell line was used to study the effect of ETS on the life span of mice with ascites HCC. Ascites in mice treated with ETS was significantly decreased. A dose-dependent prolongation of life span was observed in treated mice as compared with controls (*P<0.01*) as shown in Figure 2c. The longest survival times of mice at dosage of 1.5 mg/kg/d, 0.5 mg/kg/d and 0 mg/kg/d (control) with ETS were 34 days, 26 days and 18 days, respectively; and the mean prolonged rate of life span was 74.2% (at 1.5 mg/kg/d) and 23.2% (at 0.5 mg/kg/d).

### The toxicity of ETS is very low

Acute toxicity test indicated that the toxic effects of ETS on mice were very low. Some symptoms, including ruffled fur, tremors and hyperactivity were observed in mice treated with ETS, especially in the maximum dosage group. All 10 mice in the 4640 mg/kg group died, and 4 mice in the 2150 mg/kg group died, but there were no death in the 1000 mg/kg, 464 mg/kg and control groups. The LD_50_ of ETS was 2329.9 mg/kg, with a 95% dependable limit of 1846.7–2939.0 mg/kg.

### Molecular basis that ETS induces cancer cell apoptosis and inhibits proliferation

**Figure 3:**
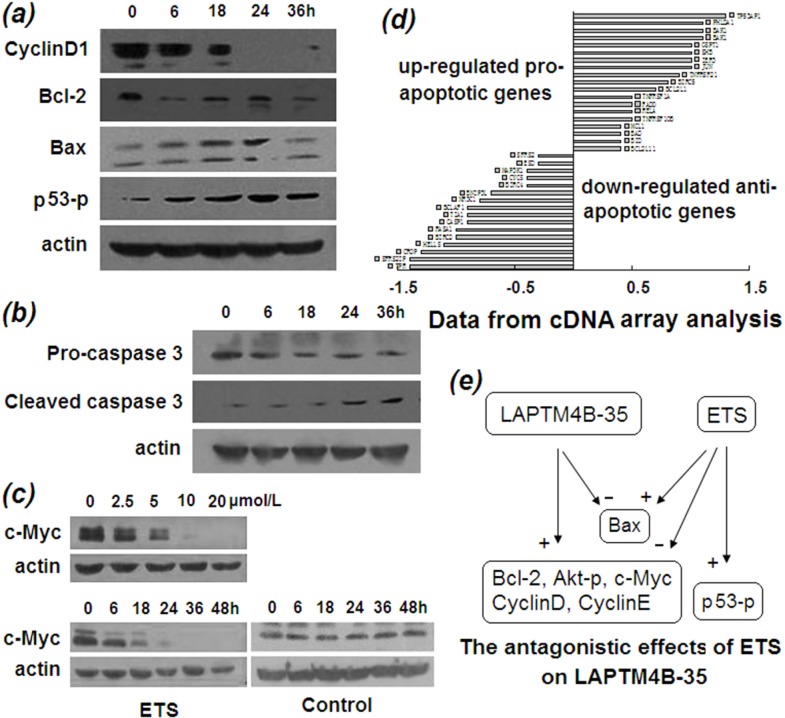
Molecular alterations induced by ETS **a.** Western blot profiles of cyclin D1, Bcl-2, Bax, and phosphorylated p53 proteins from lysate of HepG2 cells incubated in the presence of ETS (2 μmol/L) for indicated times, indicating that proliferation- and apoptosis-related proteins are altered by ETS in a time-dependent manner. **b.** Western blot profile of procaspase 3 and cleaved caspase 3 from lysate of HepG2 cells incubated in the absence and presence of ETS (2 μmol/L) for indicated times, indicating the activation of key effector molecule by ETS in apoptotic pathway. **c.** Western blot profile of c-Myc protein from lysate of HepG2 cells incubated in the absence and presence of ETS at indicated concentrations for indicated hours, indicating remarkable decrease of c-Myc protein with ETS treatment in a dose- and time-dependent manner. **d.** cDNA array analysis shows the upregulated and downregulated genes that promote and inhibit apoptosis, respectively, by treatment of ETS. **e.** Schematic representation of antagonistic effects of ETS on gene expression that had been induced by overexpression of LAPTM4B-35 in HCC.

The molecular basis that ETS induces cancer cell apoptosis and inhibits proliferation was provided by Western blot analysis. As Figure 3a shown, the antiapoptotic protein Bcl-2 were decreasing and the proapoptotic protein Bax were increasing with prolonged time of ETS treatment. Also, the critical effector molecule, caspase 3, of the apoptosis pathway was transformed by ETS from procaspase into cleaved active caspase in a time-dependent manner (Figure 3b). In addition, the p53 phosphorylation at Ser_15_ was also increasing with prolonged ETS treatment time (Figure 3a), suggesting that ETS likely stabilizes p53 protein, the critical apoptosis regulator. At the same time, cDNA array analysis showed that a large number of proapoptotic genes were upregulated and a large number of antiapoptotic genes were down-regulated by ETS treatment (Figure 3d).

Decrease of the antiapoptotic protein Bcl-2 together with the increase in proapoptotic protein Bax and cleaved caspase 3 are the molecular basis for apoptosis induced by ETS. These results also indicate the reversed roles played by ETS against the effects resulted from LAPTM4B-35 overexpression in HepG2 cells. Given that *p53* is a pivotal tumor suppressor gene, and the amount and/or function of p53 protein is decreased in more than 50% of HCC's. Functionally, *p53* regulates the transcription of genes required for cell-cycle arrest and apoptosis. In response to DNA damage, p53 protein is phosphorylated at Ser_15_ and becomes stabilized upon disruption of its interaction with its negative regulator MDM2 [20]. Figure 3a showed that ETS treatment resulted in a time dependent increase of phosphorylation of p53 Ser_15_ in HepG2 cells, indicating that ETS most likely induces apoptosis and cell-cycle arrest in HepG2 cells of LAPTM4B-35 overexpressing through promoting Ser_15_ phosphorylation in p53. In addition, expression of cyclin D1 (Figure 3a) and its transcription factor c-Myc (Figure 3c) were inhibited by ETS in a time-dependent manner, indicating not only the molecular bases for inhibition of proliferation induced by ETS, but also the antagonistic effects against overexpression of LAPTM4B-35 [11].

### LAPTM4B-35 is the target mechanistically involved in the inhibitory/killing effect of ETS on HCC cells

**Figure 4:**
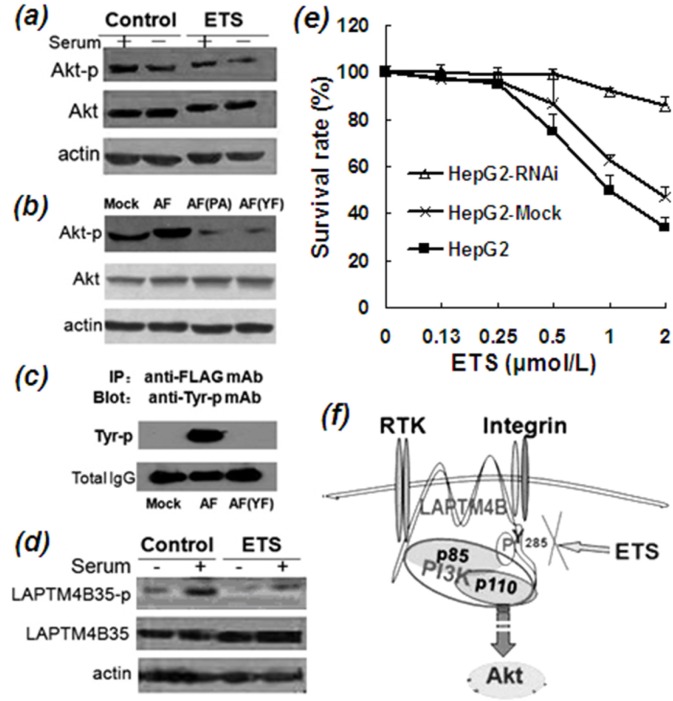
Inhibitory effects of ETS on phosphorylation of Akt and LAPTM4B-35 **a.** Western blot profile of phosphorylated Akt (Ser_473_) from lysate of HepG2 cells incubated in the absence and presence of ETS (2 μmol/L), indicating the inhibitory effect of ETS on activation of PI3K/Akt signaling pathway either in the presence or absence of serum. **b.** Western blot profile demonstrated that Akt-p (Ser_473_) is decreased in the mutated AF (PA) cells in which P12, 13, 15A mutated plasmids of LAPTM4B-35 were transfected, and also in the mutated AF (YF) cells in which Tyr (Y)_285_ Phe (F) mutated plasmids of LAPTM4B-35 were transfected, as compared with wild-type LAPTM4B-35 (AF). These results indicate that the proline-rich domain (PPRP) in the N-terminal and the Tyr_285_ in C-terminal tails of LAPTM4B-35 are necessary for Akt phosphorylation. **c.** Co-IP and Western blot profile shows that Tyr_285_ in C-terminal tails of LAPTM4B-35 is the only one Tyrosine that can be phosphorylated. BEL-7402 HCC cells were transfected by AF plasmids which contains the wild type LAPTM4B-35 cDNA with a FLAG as a tag or by AF (YF) plasmid which contains a Tyr (Y)_285_ Phe (F) mutation of LAPTM4B-35 cDNA with a FLAG as a tag. Cells were first serum-starved for 16 h, and then seeded onto laminin substrate for 15 minutes. The expressed exogenous LAPTM4B was immunoprecipitated by Anti-FLAG-mAb. After PAGE the Anti-phosphorylated Tyr mAb was used for blotting the Tyr-phosphorylated LAPTM4B. The Western blot profile shows that the phosphorylated Tyr appeared merely in the wild type BEL-7402 HCC-AF cells as one thick band, but no any Tyr-phosphoralated band appeared in the Tyr _285_ mutated BEL7402 HCC-YF cell lysate, indicating that LAPTM4B-35 Tyr_285_ is the only single phosphorylation site. **d.** Co-IP and Western blot profile shows that ETS significantly decreased the phosphorylation of LAPTM4B-35 Tyr_285_. HepG2 cells were first serum-starved for 16 h, then serum and ETS or PBS (control) were added for 15 min incubation. The cell lysate was first precipitated by anti-LAPTM4B-N10-pAb, which reacts specifically with LAPTM4B-35. Anti-phosphorylated Tyr-mAb was used as the blotting antibody. The profile shows that the phosphorylated LAPTM4B-35 is attenuated by ETS treatment compared with the control. **e.** HepG2 cells were transfected with LAPTM4B-shRNA or Mock plasmids. The expression of LAPTM4B-35 was significantly reduced in the LAPTM4B-shRNA stably transfected HepG2 cells (HepG2-RNAi) as compared with HepG2 Mock cells and the parent HepG2 cells. Cells in the three groups were respectively treated by ETS at indicated concentrations for 48 h. The HepG2-RNAi cells showed less sensitive to ETS. **f.** Schematic representation of molecular mechanism of ETS for targeting LAPTM4B-35.

Initial results showed that the inhibitory/lethal effect of ETS on HCC cells is likely correlated with LAPTM4B-35 expression levels, and we therefore further explored the involvement of LAPTM4B-35 in the effects of ETS with RNA interference (RNAi) experiments. Endogenous LAPTM4B-35 expression was silenced with shRNA transfection in HepG2 cells as in our previous study [11]. The inhibitory effects of ETS on growth of HCC cells, in which LAPTM4B-35 expression was downregulated by RNAi, were markedly reduced at ETS concentrations of 0.5, 1.0 and 2.0 μmol/L, as compared with the control Mock cells (Figure 4e). This result further suggested that the inhibitory/lethal effect of ETS on HepG2 cells is associated with LAPTM4B-35 overexpression.

Our previous study found that a number of oncoproteins that promote cell proliferation and/or resistance to apoptosis is increased in LAPTM4B-35 upregulated HepG2 cells, and is decreased in LAPTM4B-35 knocked down cells [11, 15]. The effects of ETS on the expression of these oncoproteins were further evaluated. Western blot analyses showed that all of the molecular alterations in HepG2 cells that are induced by LAPTM4B-35 overexpression were reversed by ETS (Figure 3e), including significant decreases in c-Myc (Figure 3c), cyclinD1, and Bcl-2 (Figure 3a) and increases in Bax, phosphorylated p53 (Figure 3a) and cleaved caspase 3 (Figure 3b). These results strongly suggest that ETS has functionally antagonistic effects to LAPTM4B-35. Given that *c-myc* is commonly overexpressed in HCC [21]. It is localized at 8q24 nearby the *LAPTM4B* gene (8q22). These two driver oncogenes are amplified together in breast cancer [13] and HCC (unpublished data), and both promote drug resistance [12, 13]. We found previously that c-Myc protein is significantly increased when LAPTM4B-35 expression is upregulated in HCC cells, and *vice versa* [11], indicating that c-Myc is functionally linked with LAPTM4B-35. Moreover, the accumulation of c-Myc induced by LAPTM4B-35 upregulation results from enhanced degradation through activation of PI3K/AKT/GSK3βsignaling pathway which inhibits phosphorylation of c-Myc protein thr_58_, but not mRNA transcription [11]. Herein we found that ETS treatment induced a time- and dose-dependent reduction in c-Myc (Figure 3c), suggesting the possibility of mechanical involvement of LAPTM4B-35 in ETS inhibitory effects on cancer cells.

It is well known that the PI3K/Akt signaling pathway plays a central role in antiapoptosis and cell survival in a large number of cancers and is thus considered to be a target for cancer therapy [22]. Phosphorylated Akt (Ser_473)_ (Akt-p)) is commonly recognized as a marker for activation of the PI3K/Akt signaling pathway. Our previous studies demonstrated that the PI3K/Akt/GSK3β signaling pathway is over activated by LAPTM4B-35 overexpression [11, 12, 15]. In this study the effect of ETS on the PI3K/Akt signaling pathway was evaluated. We found that Akt-p (Ser_473_) is significantly reduced in ETS-treated HCC cells (Figure 4a), indicating ETS has an inhibitory effect on activation of the PI3K/Akt signaling pathway. The mechanism for this effect of ETS was then studied. Given that PI3K consists of two subunits, including the p110 catalytic subunit and the p85α regulatory subunit. The kinase activity of PI3K p110 is normally inhibited by binding of p85α. We found that LAPTM4B-35 can interact/bind with p85α by two motifs. One motif is the proline-rich motif (PPRP) in the N terminus [10], and the other is phosphorylated Tyr_285_ in the C-terminus of LAPTM4B-35 (Figure 4b, previously performed by Xuanhui Wei in our Lab). Based on the interaction/binding between LAPTM4B-35 and PI3K p85α, the inhibitory effect of p85α on p110 is abolished and downstream Akt is consequently phosphorylated and activated [12]. We also found that LAPTM4B-35 Tyr_285_ is the sole tyrosine that is phophorylable in LAPTM4B-35 molecule (Figure 4c, previously performed by Hua Yang in our Lab). In the current study, the effect of ETS on LAPTM4B-35 Tyr_285_ phosphorylation was evaluated with Co-IP and Western blot analysis. The results showed that ETS significantly attenuated the phosphorylation of LAPTM4B-35 Tyr_285_ (Figure 4d) either in the presence or absence of serum, as compared with the control. This suggests a possible mechanism, by which activation of the PI3K/Akt signaling pathway is attenuated by ETS through inhibition of the interaction of LAPTM4B-35 and PI3K p85α (Figure 4f).

Taken together, it is suggested that the inhibitory/lethal effect of ETS on HCC cells is LAPTM4B-35 dependent, in other words inhibitory/lethal effect of ETS on HCC cells is based on targeting LAPTM4B-35.

### cDNA arrays show that ETS upregulates the expression of proapoptotic genes and downregulates the expression of antiapoptotic genes

cDNA arrays showed that ETS treatment (2 μmol/L, for 24 hours) results in a remarkable alteration of gene expression profiles. Genes that were downregulated more than 2 fold are mainly genes involved in antiapoptosis, cell cycle arrest, and cell signaling transduction. Genes that were upregulated more than 2 fold are mainly genes involved in promotion of apoptosis and response to inflammation (data not shown). These results provide further evidence that ETS significantly impacts the cell cycle, apoptosis and signaling transduction.

### Conclusion

ETS induces apoptosis and inhibits growth and metastasis of human HCC xenograft in nude mice through targeting LAPTM4B-35 protein, and is thus a promising candidate for targeted therapy of HCC.

## MATERIALS AND METHODS

### Ethics statement

Investigation has been conducted in accordance with the ethical standards and according to the Declaration of Helsinki and according to national and international guidelines and has been approved by the authors’ institutional review board.

### Cell lines and animals

In this study two LAPTM4B-35 overexpressing wild type human hepatocellular carcinoma cell lines, (HepG2 and Bel-7402 cell line) and a LAPTM4B-35 knock down HepG2 cell line (HepG2-RNAi) were applied. All of the cell lines used were maintained in our laboratory. Cells were cultured in medium of DMEM supplemented with 10% Newborn Calf Serum (NCS) at 37°C 5%CO_2_. SPF ICR mice and BALB\c-nude mice were provided by Department of Laboratory Animal Science, Peking University.

### Survival rate analysis

The survival rate of cells was measured by counting the living cells number using acid phosphatase assay (APA) [23] in the presence and absence (control) of ETS at indicating concentrations for 48 hours. The color development was measured at 405 nm using a rapid microplate reader. The OD value was directly proportional to the number of living cells in each well. Growth rate = [(ODtreated – ODzero) / (ODcontrol – ODzero)] x 100%.

### LIVE/DEAD* viability/cytotoxicity kit assay for detection of viable and dead cells

Cells (1 × 10^4^) were seeded in 96-well plates. The cells were then fluorescently double stained with Calcein-AM (1 μmol/L) and EthD-1 (2 μmol/L) reagent in LIVE/DEAD Viability/Cytotoxicity Kit (Invitrogen, USA) and observed under a fluorescence microscope after 30 min incubation at 37°C [24]. The cells emitting green fluorescence were alive which were only stained by Calcein-AM and the cells emitting red fluorescence were dead or apoptosis at middle and late phase which were only stained by EthD-1.

### Screening of effective anticancer compounds

Cells (3000/well) were seeded in 96-well plate and cultured for 24 h, and then followed by exposing to a chemical for 48 h. The curves of cell survival rate were drawn according to the survival rate measured by APA at indicated concentrations or times. The HepG2 and Bel-7402 cell lines were used in first screening on 1697 compounds at a concentration of 50 μmol/L. The effective compounds that reduce cell survival rates were selected for further screening at a series of concentrations from 0.1 to 100 μmol/L. ETS, as one of the most effective compounds was selected for further study. In order to verify the universal effect of ETS on various cancer cells, HepG2, Bel-7402, HeLa, HLE, H22 and fetal liver cell lines (FLC, which was a normal cell line) were used in the following experiments. The concentrations of ETS was 0, 0.25, 0.5, 1.0, 2.0, 4.0 μmol/L. Cisplatin, Doxorubicin, Mitomycin and 5-Fluorouracil (provided by the third hospital of Peking University) were used as positive controls with concentrations of 0.8, 1.6, 3.2, 6.3, 12.5, 25, 50 μmol/L.

### Flow cytometric analysis for apoptosis

Cells (2 × 10^5^) were seeded in 6 well plates for 0, 8, 16, 24, 36 hours in the presence (2 μmol/L) and absence of ETS. Both adherent and floating cells were collected. Apoptosis was analyzed using an Annexin V Apoptosis Detection kit (Biosea, Beijing, China) with a FACSCalibur flow cytometer (Becton Dickinson).

### Detection of ETS effects on human Bel-7402 HCC xenograft *in vivo*

Healthy female BALB\c-nude mice (n=56, weight 18-22g) were subcutaneously inoculated with human HCC Bel-7402 cells (1 × 10^6^) at the foreside on back, then stochastically divided into 7 group: ETS (22.5, 7.5, 2.5 mg/kg/d), cisplatin (1.0 mg/kg/d), mitomycin (1.0 mg/kg/d), solvent control and blank control (PBS), respectively. Each group contained 8 mice. ETS, cisplatin, mitomycin were intraperitoneally administered by injection from the day 9 after inoculation of Bel-7402 cells when the tumor grew out. Tumor volume was measured twice a week. Tumor volume=1/2 × tumor length × tumor width^2^. Inhibitory rate of tumor = (mean tumor weight of control - mean tumor weight of treated mice) / mean tumor weight of control × 100%.

### Life span detection of mouse with H22 ascites hepatocarcinoma

Healthy ICR mice (n=30, weight 18~22g) were chosen and peritoneally inoculated with H22 cells (1 × 10^6^), a mouse hepatocarcinoma line, then divided into 3 groups: high dose ETS (1.5 mg/kg/d), low dose ETS (0.5 mg/kg/d) and control (NS). Each group contained 10 mice (5 male and 5 female). ETS were intraperitoneally administered by injection from the third day after H22 cells inoculation. Abdominal girth was measured for every 4 days and life span was also tracked record lastly. Prolong rate of life span = (mean life span of treated mice – mean life span of control) / mean life span of control × 100%.

### Western blot analysis

Cells were lysed in prechilled RIPA lysis buffer (Pierce Biotechnology, Rockford, IL) containing protease inhibitor cocktail (Roche, Basel, Switzerland). For detection of phosphorylation in signaling proteins, cells were serum deprived for 16 h, then stimulated with 20% fetal bovine serum (FBS) for 10 min, and phosphatase inhibitor cocktail (Roche) was added to the lysis buffer. Western blot was carried out as previously described with appropriate antibodies [14], including anti-LAPTM4B-N10-pAb (produced by our lab); anti-Bcl-2, anti-Bax, anti-phospho-tyrosine protein, anti-actin (all from Santa Cruz Biotechnology); anti-Akt, anti-cyclinD1, anti-phospho-p53 (Ser_15_), anti-caspase3, anti-cleaved caspase3, and anti-phospho-Akt kinase (Ser_473_) (all from Cell Signaling Technology).

### Co-Immunoprecipitation (Co-IP)

Co-IP was used to identify the phosphorylation of LAPTM4B-35. HepG2 cells were treated with ETS (2 μmol/L) for 24 h. 10% FCS was present in the medium of the first 8 hours of ETS treatment, and was removed in the following 16 hours of ETS treatment. After activation by fibronectin substrate and/or FCS for 15 min, the HepG2 cells were harvested and lysed with membrane lysis buffer. LAPTM4B-35 in HepG2 cell lysate was precipitated by anti-LAPTM4B-N10-pAb. After isolated by Protein G/A Agarose beads, the LAPTM4B-35-N10-pAb immuno-precipitant was subjected to PAGE. Then anti-phosphorylated Tyr-antibody was applied to blot the phosphorylated LAPTM4B-35 that was precipitated by anti-LAPTM4B-N10-pAb and separated by PAGE.

### cDNA array analysis

The cDNA array was used to detect the gene expression spectrum induced by ETS. HepG2 cells exposed to ETS (2 μmol/L) for 24 h were gathered by adding a reagent of Trizol (Invitrogen) into flask after having been rinsed with PBS. The next steps were managed with GeneChip Human Genome U133 Plus 2.0 (Affymetrix Inc Company) and completed by Shanghai Jingtai Biotechnology Company.

### Acute toxicity analysis

Trimmed spearman-karber method was used to determine the toxicity of ETS in the acute toxicity test. ETS was orally gavage administered by once as the mice being fasting overnight. The mice were monitored for morbidity and mortality for 21 days, and lastly the median lethal dose (LD_50_) and 95% confidence interval was determined by the number of dead mice.

### Statistical analysis

Statistical significance was analyzed using Student's *t* test, Kruskal-Wallis or a one-way analysis of variance (ANOVA) and *P<0.05* was considered significant.
